# Study on the correlation between Helicobacter Pylori and biological characteristics of early Gastric Cancer

**DOI:** 10.7150/jca.46392

**Published:** 2021-01-21

**Authors:** Guanqun Chao, Xinli Chen, Shuo Zhang

**Affiliations:** 1 Department of Family Medicine, Sir Run Run Shaw Hospital, Zhejiang University, China.; 2 Department of Gastroenterology, The First Affiliated Hospital, Zhejiang Chinese Medical University, China.

**Keywords:** helicobacter pylori, early gastric cancer, biological characteristics

## Abstract

**Objective:** Retrospective analysis was used to determine the population diagnosed with EGC, and HP infection was used as the cut-off point to further evaluate the correlation between* helicobacter pylori* (HP) infection and tumor biological characteristics of early gastric cancer (EGC).

**Methods:** All cases were collected from patients diagnosed with EGC through endoscopic surgery or surgical procedures from January 2009 to October 2018. General information, tumor site, tumor pathology, HER2 immunohistochemical results, and degree of HP infection were collected for retrospective analysis.

**Results:** A total of 111 cases were collected in this study. Among the HP negative group, there were statistically significant differences in tumor sites between the uninfected group and the previously infected group (*P*<0.05).There were significant differences in monocyte infiltration and neutrophil infiltration between the positive and negative groups (*P*<0.05).The differentiated adenocarcinoma in the positive group was significantly lower than that in the negative group. The incidence rate of Mixed type cancer was significantly higher than that in the positive group (*P*<0.01). In the positive group of HP, there was a statistically significant difference in HER2 between the unsterilized group and the previously sterilized group (*P*<0.05).There was a statistically significant difference in HER2 between the HP positive group and the HP negative group (*P*<0.01). HP infection was significantly correlated with HER2 index and presented a positive correlation (*P*=0.014).

**Conclusion:** HP infection is related to the tumor site and mucosal inflammatory infiltration of EGC. The malignant degree of EGC complicated with HP infection is higher, and most of them are mixed type. The degree of HP infection was positively correlated with the degree of invasion and malignancy of ECG. Furthermore, the tumor indicator HER2 is closely related to HP infection, and the detection of HP combined with HER2 is of great significance in the discovery of EGC and the evaluation of its malignancy.

## Introduction

Gastric cancer is still the fifth most common malignancy in the world and is believed to be the third leading cause of cancer death 1. Gastric cancer (GC) accounts for about half of the world's new cases each year in east Asia, especially in China, Japan and South Korea 2. Early gastric cancer (EGC) refers to GC with tumor infiltration confined to the mucosa or submucosa, regardless of lymph node metastasis 3. The study found that EGC accounted for a large proportion of GC 2. Because GC has no obvious clinical manifestations and significant imaging features, the diagnosis of EGC is often delayed 4. Although the diagnosis and treatment of cancer have been developed with the progress of technology, the prognosis of advanced GC is still very poor.As we all know, malignant tumor metastasis is a major challenge for treatment 5. Therefore, the prevention of GC, early identification and early treatment, are keys to reduce the death rate of GC.

Helicobacter pylori (HP) is a gram-negative bacillus, which is fixed in the gastric mucosa, causing chronic inflammation 6. The bacteria are presented in most people's gastric mucosa and can cause diseases such as gastritis, ulcers, cancer and lymphoma 7. HP was considered as a class I carcinogen by the World Health Organization (WHO) 8. Compared with uninfected people, the incidence of gastric cancer in patients with HP infection was significantly higher 9. However, the eradication rate of HP also decreased significantly due to the increase of drug resistance rate 10. Scholars believe that the incidence of GC in developing countries is significantly increased due to the increase of HP infection rate and related health and environmental problems 11. At present, there are many studies on the correlation between HP infection and GC, but few studies on the correlation between the biological characteristics of EGC. Whether there is a difference in the occurrence of EGC between patients who infected with HP without sterilization and those who reinfected after sterilization and difference between uninfected patients and previously infected patients who had been eradicated from HP remain to be further confirmed. In this study, retrospective analysis was used to determine the population diagnosed with EGC, and HP infection was used as the cut-off point to further evaluate the correlation between HP infection and tumor biological characteristics of EGC.

## Methods

### Inclusion criteria

All cases were collected from patients diagnosed with EGC through endoscopic surgery or surgical procedures from January 2009 to October 2018.The criteria for the diagnosis of EGC refer to the Chinese consensus on EGC screening and endoscopic diagnosis and treatment (Changsha, April 2014): The carcinoma of EGC is limited to gastric mucosa or submucosa, with or without lymph node metastasis. Age: 18-70.

### Exclusion criteria

- Patients with GC who underwent endoscopic or surgical treatment from 2009 to 2017 and whose pathological results showed positive margins of cancer tissue or infiltration depth beyond the submucosa;
- Patients with esophageal cancer;
- Patients with serious primary diseases such as heart, liver, kidney, brain and hematopoietic system;
- Have a history of other malignant tumors;
- Incomplete information;
- Family history of cancer.

### Case grouping

- Group A (Unsterilized group): HP positive patients who had never been sterilized;
- Group B (Previously sterilized group): HP positive patients who had been sterilized but reinfected;
- Group C (Uninfected group): HP positive patients who had never been infected;
- Group D (Previously eradicated group): HP negative patients who had previously been eradicated from *helicobacter pylori.*

### Case data collection

The basic information of all selected cases was subject to the case record, the location and size (maximum diameter) of the tumor were subject to the gastroendoscopy report.

The pathological report prevailed in the pathological results of gastroendoscopy, including:

(1) Infiltration depth: mucosa; muscularis mucosa; submucosa.

(2) Histological type: early gastric cancer can be divided into the following histological types according to the criteria of Japanese gastric cancer classification scheme: Papillary adenocarcinoma; tubular adenocarcinoma (highly differentiated; intermediate differentiation; poorly differentiated adenocarcinoma; signet ring cell carcinoma; mucinous adenocarcinoma; mixed adenocarcinoma.

(3) Differentiation degree: according to WHO gastric cancer classification.

- Differentiated: papillary adenocarcinoma; tubular adenocarcinoma (highly differentiated; the differentiation; low differentiation).
- Undifferentiated: signet ring cell carcinoma, mucinous adenocarcinoma, mixed adenocarcinoma.

(4) HER2 immunohistochemical results: according to the pathological report.

- Negative: immunohistochemical results were 0 and 1+, or 2+ additional Fisher test was negative;
- Positive: immunohistochemical results were 3+ or 2+ additional Fisher test was positive.

(5) The gastritis classification was determined by the Update Sydney system.

(6) Helicobacter pylori classification.

Histopathological information of early gastric cancer was extracted from the pathological database, and the pathological specimens stained by w-s silver staining were graded for* h. pylori.*

- HP -: helicobacter pylori was not found on the special stain;
- hp1+: occasionally or less than 1/3 of the total length of the specimen, a small number of helicobacter pylori;
- hp2+: the distribution of helicobacter pylori is more than 1/3 of the whole sample, but less than 2/3, or continuous, thin and sparse, exists in the full length of the sample
- hp3+: helicobacter pylori was distributed in heaps throughout the whole length of the specimen.

### Statistical analysis

The measurement data were expressed as mean standard deviation (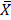
±SD), and spss25.0 statistical software package was used for statistical analysis. Chi-square test was used for the comparison of classified data between groups, one-way ANOVA was used for the comparison of means between groups, and LSD method was used for pair-wise comparison between groups. Independent sample t test was used for sample mean. Spearman method was used for correlation analysis of grade data. *P* < 0.05 was considered statistically significant.

## Results

### General data analysis

A total of 111 cases were collected in this study, including 47 cases in the helicobacter pylori positive group (35 cases in the unsterilized group and 12 cases in the previously sterilized group) and 64 cases in the helicobacter pylori negative group (37 cases in the uninfected group and 27 cases in the previously infected group). The proportion of males and females and the age range of each group are shown in **Table 1**.

### Endoscopic data analysis

Endoscopic data of each patient: tumor site and maximum tumor diameter are shown in **Table 2**. Among the HP negative group, there were statistically significant differences in tumor sites between the uninfected group and the previously infected group (*P*<0.05). Compared with the previously infected group, the uninfected group was more likely to be in the upper two-thirds of the stomach. There was no statistically significant difference in tumor sites between the unsterilized group and the previous sterilization group, and between the HP positive group and the negative group (*P*>0.05). There was no significant difference in tumor size among groups (*P*>0.05).

### Gastritis classification

Pathological data of each case: see **Table 3** for the classification of gastritis. There were significant differences in monocyte infiltration and neutrophil infiltration between the positive and negative groups (*P*<0.05). There was no significant difference in atrophy and intestinal metaplasia between the positive and negative groups. There was no significant difference in gastritis grade between the unsterilized group and the former sterilized group, and between the uninfected group and the former infected group.

### Histological type

**Figure 1:**
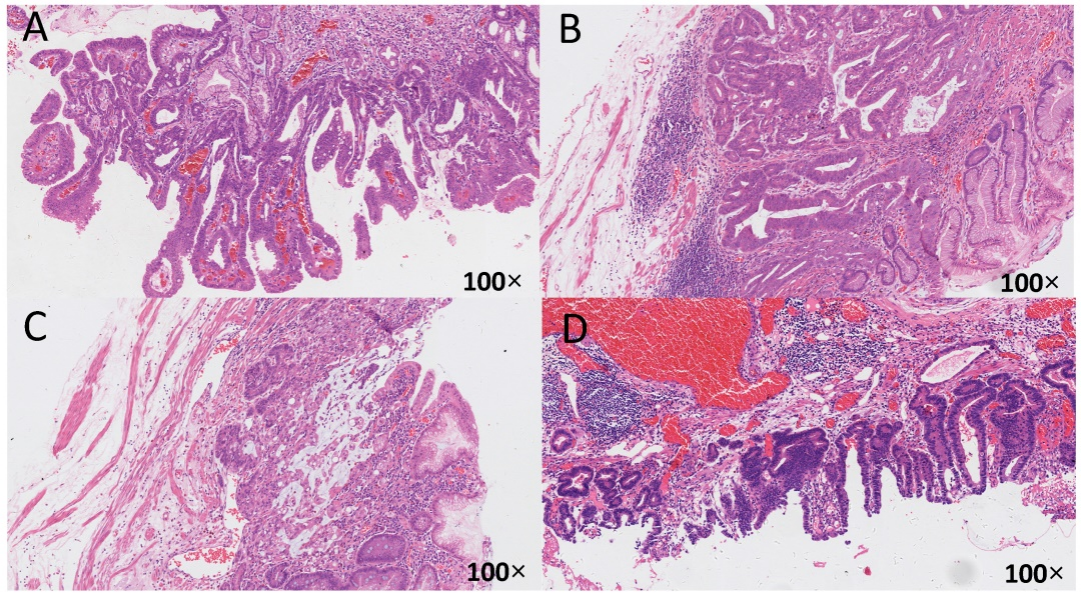
** The relationship between HP infection and early gastric cancer differentiation.** The histological specimens were all stained with H-E, and the magnification is 100 times. A: HP (-), from uninfected group, differentiated type, highly differentiated adenocarcinoma, infiltrate into the mucosa; B: HP (2+), from unsterilized group, mixed type, moderately poorly differentiated adenocarcinoma and mucinous adenocarcinoma, infiltrate into the submucosa; C: HP (2+), from previously sterilized group, differentiated type, moderately differentiated adenocarcinoma, infiltrate into the submucosa; D: HP (3+), from previously sterilized group, differentiated type, moderately differentiated adenocarcinoma, infiltrate into the submucosa.

The correlation between different helicobacter pylori infection history and eradication history and the histological types of early gastric cancer is shown in **Table 4**, and the histological pattern was illustrated in **Figure 1**. The differentiated adenocarcinoma in the positive group was significantly lower than that in the negative group. Mixed type cancer was significantly higher in the positive group than in the negative group (*P*<0.01). There was no significant difference in histological types of early and middle gastric cancer between the other groups (*P*>0.05).

### The relationship between tumor indicator HER2 and early gastric cancer

The relationship between tumor indicator HER2 and EGC differentiation was shown in Table 5, the relationship between tumor indicator HER2 and EGC infiltration was shown in **Table 6**. The results shown that the rate of EGC infiltrated to submucosa in HER2 positive group was significantly higher than HER2 negative group (*P*<0.05). There was no significant difference in histological types of early and middle gastric cancer between the other groups (*P*>0.05). There was no significant difference in rate of EGC infiltrated to mucous or muscularis mucosa between HER2 positive group and HER2 negative group (*P*>0.05).

### Association between HP and tumor marker HER2 in early gastric cancer

**Figure 2:**
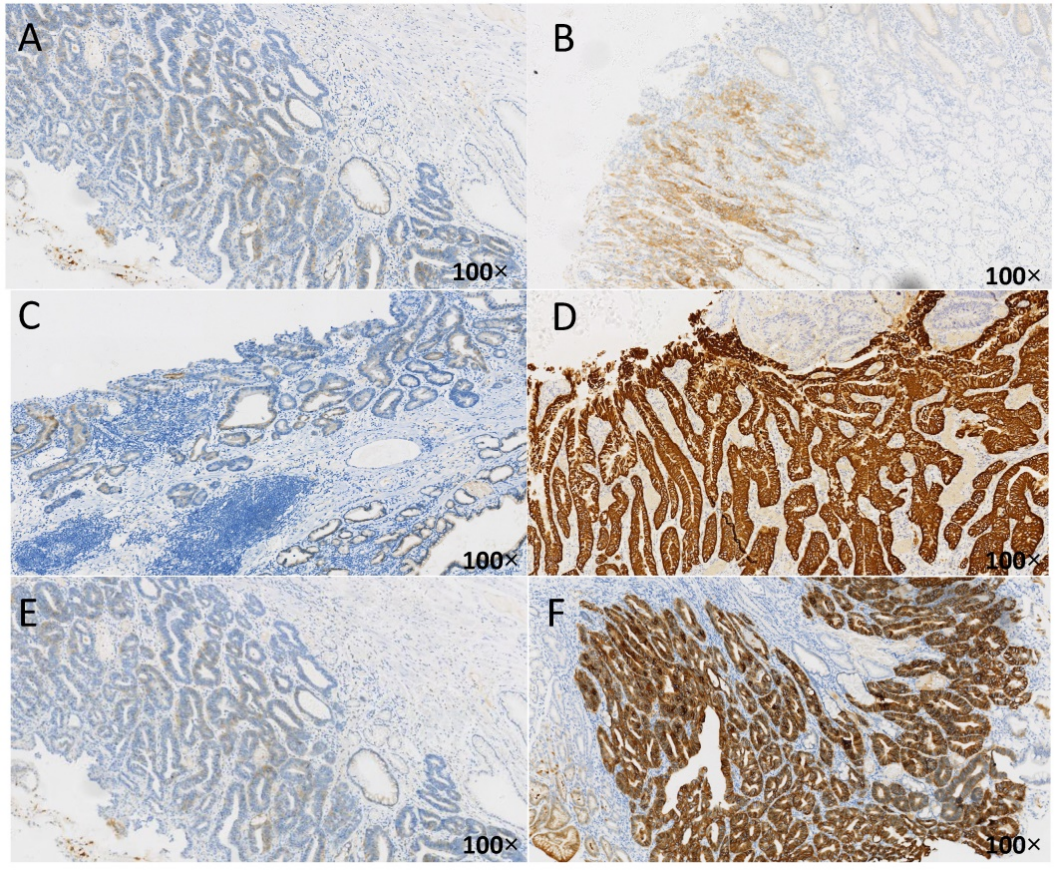
** Immune-histochemical results of HER2 in different cases of HP infection.** The brown-yellow particles in the figure represent the expression of HER2. A: HER2 negative (1+), from uninfected group, HP(-); B:HER2 positive (2+), from unsterilized group, HP(1+); C: HER2 negative (1+), from previously sterilized group, HP (2+); D: HER2 positive (3+), from unsterilized group, HP (2+); E: HER2 negative (1+), from previously sterilized group, HP (3+); F: HER2 positive (3+), from unsterilized group, HP (3+).

The influence of different HP infection history and eradication history on early gastric cancer HER2 is shown in **Table 7**, and the immunohistochemical results of HER2 are shown in **Figure 2**. In the positive group of HP, there was a difference in HER2 between the unsterilized group and the previously sterilized group (*P*<0.05). There was a significant difference in HER2 between the HP positive group and the HP negative group (*P*<0.01). There was no significant difference between the uninfected group and the previously infected group. And as shown in **Figure 2**, compare with the HP negative group, the expression of HER2 in HP positive group obviously higher. What's more, the expression of HER2 in unsterilized group obviously higher than previously sterilized group.

### Linear relationship between HP infection and HER2

According to the correlation analysis, HP infection was significantly correlated with HER2 index and presented a positive correlation (*P*=0.014) (**Table 8**).

## Discussion

Gastric cancer has been recognized as a global health problem with a high mortality rate. HP infection was associated with the majority of gastric cancer, with a conservative estimate of 74.7% 12. Therefore, as early as 1994, the world health organization (WHO) listed HP as a class I carcinogen, and the international agency for research on cancer (IARC) also listed HP as a class I carcinogen. In recent years, the incidence of gastric cancer tends to decline 13, which may be related to the reduction of smoking, change of lifestyle, reduction of HP infection and increase of HP eradication rate 14. However, due to the extension of life expectancy and the increase of population, although the death rate of gastric cancer decreased, the number still showed a state of flat or even increased 15. Early detection and treatment of GC can significantly reduce the mortality of GC, so this study aims to study the correlation between the biological characteristics of EGC and HP infection.

Our study shows that there is no statistical difference in general data between the four groups, indicating that there is comparability among the four groups. For the HP negative group, there were significant differences in tumor sites between the uninfected group and the previously infected group. There was no significant difference in tumor size among groups. Thus, it can be seen that patients without HP infection still have the possibility of GC, but the location of the tumor is more inclined to the upper two-thirds of the stomach. However, HP infection does not affect the size of EGC. In addition to HP infection, lifestyle, genetic changes and family history were all risk factors for GC 16. Therefore, HP infection is not an independent risk factor for GC. Although family history has been excluded from our study, it is impossible to make a precise distinction about lifestyle. In future studies, we expect to further stratify the design of lifestyle. GC usually occurs in the lower third of the stomach, according to a SouthKorean study 17. A US study suggested that 32.2% of EGCs were located in the antrum, while both early and late cancers in South Korea were more concentrated in the antrum 18. However, our study did not suggest differences in tumor site in the HP-positive group, but it was found that in the HP-negative group, the tumors were more concentrated in the upper two-thirds of the stomach in the uninfected group, which was similar to other studies.

In our study, there were significant differences in monocyte infiltration and neutrophil infiltration between the positive and negative groups, and there was no significant difference in atrophy and intestinal metaplasia between the positive and negative groups. It is well known that atrophy, intestinal metaplasia, and gastric mucosal dysplasia are precancerous lesions of GC 19. Our study indicated that the incidence of precancerous lesions was not abnormal between the two groups regardless of whether HP infection was present, which may be related to the fact that all the included patients were patients with EGC and had precancerous lesions. Studies have suggested that HP infection is associated with gastric cancer; however, gastric cancer is rare in gastric mucosa without inflammation 20. In our study, there was a significant increase in infiltration of gastric mucosal inflammatory cells in the HP infected group, which was consistent with the scholars' study.

According to the presence or absence of tubular structures, gastric cancer is currently divided into differentiated and non-differentiated types 21. Our study found that among the patients with EGC, HP-positive patients were mainly mixed type, and HP-negative patients were mainly differentiated type. Scholars have proposed that mixed type of EGC is an independent risk factor for lymph node metastasis, which is more likely to occur than simple differentiated type of EGC and non-differentiated type of EGC, requiring special surgical options 22. It has always been believed that the higher the grade of atypia, the lower the degree of differentiation, the greater the malignant characteristics of EGC 23. All above indicate that HP infection may be one of the important factors that aggravate the invasive and malignancy of EGC.

Human epidermal growth factor receptor 2 (HER2) is a type of growth factor receptor that mediates cell growth 24. HER2 is overexpressed in 10% to 30% of GCs, and HER2-positive tumors are more aggressive and have a poorer prognosis than HER2-negative tumors 25. HER2 overexpression plays an important role in the apoptosis, proliferation and vascular growth of many solid tumors 26. The researchers noted that HER2 plays an important role in the assessment of gastric cancer, and believed that it is associated with its clinical characteristics and can be used to evaluate the prognosis 27. Our study found that the rate of EGC infiltrated to submucosa in HER2 positive group was significantly higher than HER2 negative group. On the contrary, the infiltration depth of HER2 negative group tended to be mucous and muscularis mucosa, confirming that HER2-positive EGC patients had a higher degree of malignancy and greater risk. In addition, positive rates of HER2 were higher in the HP-positive group than in the HP-negative group, while in the HP-positive group, the HER2 positive rate was higher in the never-eradicated group than in the previous eradicated group. More importantly, the positive rate of HER2 was positively correlated with the degree of HP infection. It can be seen that HP infection may aggravate the invasiveness of EGC, resulting in poor prognosis, and which is an important factor for the increase of malignancy degree of GC. The early eradication of HP can effectively reduce the positive rate of HER2, thereby reducing the malignant degree of the tumor and improving the prognosis.

To sum up, GC is a common malignant tumor with high morbidity and mortality. Because the treatment of EGC can significantly reduce the mortality and prolong the survival cycle, the diagnosis and screening of EGC are of great importance. HP infection is one of the risk factors of EGC, and can affect the degree of malignancy and invasiveness of GC. HP infection is related to the location, malignant degree and the degree of inflammatory infiltration. More importantly, the expression of tumor indicator HER2 was correlated with the degree of EGC malignancy, and the positive rate of HER2 was positively correlated with the positive rate of HP. Therefore, doing regular screening for HP infection and HP eradication as soon as possible can effectively reduce positive rate of HER2, and then reduce the incidence of GC, the malignancy of GC and the rate of mortality.

## Conclusion

HP infection is related to the tumor site and mucosal inflammatory infiltration of EGC. The malignant degree of EGC complicated with HP infection is higher, and most of them are mixed type. The degree of HP infection is positively correlated with the degree of invasion and malignancy of ECG. Furthermore, the tumor indicator HER2 is closely related to HP infection, and the detection of HP combined with HER2 is of great significance in the discovery of EGC and the evaluation of its malignancy.

## Figures and Tables

**Table 1:** Grouping and general data analysis

	HP positive (N=47)		HP negative (N=64)	
	Unsterilized group (N=35)	Previously sterilized group (N=12)	Uninfected group (N=37)	Previously eradicated group (N=27)
Number of cases	35	12	37	27
Sex ratio (male/female)	24/11	7/5	26/11	15/12
Age	62.6±6.7	65.0±7.6	63.9±10.0	64.3±8.3

There was no significant difference in gender or age among the groups (*P* > 0.05).

**Table 2:** The tumor site and size of early gastric cancer between each group

	HP positive (N=47)		*P*	HP negative (N=64)		*P*	*P*
	Unsterilized group (N=35)	Previously sterilized group (N=12)		Uninfected group (N=37)	Previously eradicated group (N=27)		
**The tumor site**							
Upper two-thirds	15	6	NS	18	5	0.013	NS
Lower third of the stomach	20	6		19	22		
Size (cm)	1.68±1.19	1.82±1.10	NS	1.42±0.93	1.49±0.91	NS	NS

Among the HP negative group, there were significant differences in tumor sites between the uninfected group and the previously infected group (*P*<0.05). There was no significant difference in tumor size between groups (*P*>0.05).

**Table 3:** Analysis of inflammation in early gastric cancer

	HP positive (N=47)		*P*	HP negative (N=64)		*P*	*P*
	Unsterilized group (N=35)	Previously sterilized group (N=12)		Uninfected group (N=37)	Previously eradicated group (N=27)		
Atrophy	21 (60%)	6 (50%)	0.53	16 (43%)	15 (55%)	0.38	0.48
Intestinal metaplasia	27 (77%)	10 (83%)	0.94	31 (83%)	23 (85%)	0.94	0.62
Mononuclear infiltration	5 (14%)	2 (17%)	0.84	2 (5%)	0 (0%)	0.22	0.025*
Neutrophil infiltration	23 (66%)	8 (67%)	0.74	19 (51%)	12 (44%)	0.60	0.025*

*There were significant differences in monocyte infiltration and neutrophil infiltration between the positive and negative groups (*P*<0.05).

**Table 4:** Differentiation of early gastric cancer

	HP positive(N=47)		*P*	HP negative(N=64)		*P*	*P*
	Unsterilized group (N=35)	Previously sterilized group (N=12)		Uninfected group (N=37)	Previously eradicated group (N=27)		
Differentiated type	26 (74.3%)	9 (75%)	NS	34 (91.9%)	23 (85.2%)	NS	0.01*
Undifferentiated type	2 (5.71%)	0 (0%)		2 (5.41%)	4 (14.8%)		
Mixed type	7 (20%)	3 (25%)		1 (2.70%)	0 (0%)		

*The differentiated adenocarcinoma in the positive group was significantly lower than that in the negative group. Mixed type cancer was significantly higher in the positive group than in the negative group (*P*<0.01). There was no significant difference in histological types of early and middle gastric cancer between the other groups (*P*>0.05).

**Table 5:** The relationship between tumor indicator HER2 and EGC differentiation

	HER2 positive (N=15)	HER2 negative (N=96)	*P*
Differentiated type	11 (73.3%)	81 (84.4%)	0.327
Undifferentiated type	1 (6.7%)	7 (7.3%)	
Mixed type	3 (20.0%)	8 (8.3%)	

There was no significant difference in histological types of early and middle gastric cancer between the other groups (*P*>0.05).

**Table 6:** The Relationship between tumor indicator HER2 and EGC infiltration

	HER2 positive (N=15)	HER2 negative (N=96)	*P*
Mucous	3 (20%)	36 (37.5%)	0.187
Muscularis mucosa	6 (40%)	46 (47.9%)	0.769
Submucosa	6 (40%)	14 (14.5%)	0.043*

*The rate of EGC infiltrated to submucosa in HER2 positive group was significantly higher than HER2 negative group (*P*<0.05).There was no significant difference in rate of EGC infiltrated to mucous or muscularis mucosa between HER2 positive group and HER2 negative group (*P*>0.05).

**Table 7:** Correlation analysis of HP and tumor marker HER2 in early gastric cancer

HER2	HP positive (N=47)		*P*	HP negative (N=64)		*P*	*P*
	Unsterilized group (N=35)	Previously sterilized group (N=12)		Uninfected group (N=37)	Previously eradicated group (N=27)		
Negative	23	12	0.049*	36	25	0.779	0.002**
Positive	12	0		1	2		

*In the positive group of HP, there was a difference in HER2 between the unsterilized group and the previously sterilized group (*P*<0.05). **There was a significant difference in HER2 between the HP positive group and the HP negative group (*P*<0.01).

**Table 8:** Linear relationship between HP infection and HER2

The correlation coefficient	1.000	.232*
***Szpilman Rho***		
**Degree of HP infection**		
Sig. (Double tail)		.014
N	111	111
**HER2**		
The correlation coefficient	.232*	1.000
Sig. (Double tail)	.014	
N	111	111

*At the level of 0.05 (double-tailed), the correlation was significant.
